# Time-Varying Effective Connectivity for Describing the Dynamic Brain Networks of Post-stroke Rehabilitation

**DOI:** 10.3389/fnagi.2022.911513

**Published:** 2022-05-24

**Authors:** Fangzhou Xu, Yuandong Wang, Han Li, Xin Yu, Chongfeng Wang, Ming Liu, Lin Jiang, Chao Feng, Jianfei Li, Dezheng Wang, Zhiguo Yan, Yang Zhang, Jiancai Leng

**Affiliations:** ^1^International School for Optoelectronic Engineering, Qilu University of Technology (Shandong Academy of Sciences), Jinan, China; ^2^School of Electrical Engineering and Automation, Qilu University of Technology (Shandong Academy of Sciences), Jinan, China; ^3^The Clinical Hospital of Chengdu Brain Science Institute, Ministry of Education Key Lab for Neuroinformation, University of Electronic Science and Technology of China, Chengdu, China; ^4^School of Life Science and Technology, University of Electronic Science and Technology of China, Chengdu, China; ^5^The Department of Physical Medicine and Rehabilitation, Qilu Hospital, Cheeloo College of Medicine, Shandong University, Jinan, China

**Keywords:** stroke, motor imagery, time-varying network, graph theory, Fugl-Meyer assessment

## Abstract

Hemiplegia is a common motor dysfunction caused by a stroke. However, the dynamic network mechanism of brain processing information in post-stroke hemiplegic patients has not been revealed when performing motor imagery (MI) tasks. We acquire electroencephalography (EEG) data from healthy subjects and post-stroke hemiplegic patients and use the Fugl-Meyer assessment (FMA) to assess the degree of motor function damage in stroke patients. Time-varying MI networks are constructed using the adaptive directed transfer function (ADTF) method to explore the dynamic network mechanism of MI in post-stroke hemiplegic patients. Finally, correlation analysis has been conducted to study potential relationships between global efficiency and FMA scores. The performance of our proposed method has shown that the brain network pattern of stroke patients does not significantly change from laterality to bilateral symmetry when performing MI recognition. The main change is that the contralateral motor areas of the brain damage and the effective connection between the frontal lobe and the non-motor areas are enhanced, to compensate for motor dysfunction in stroke patients. We also find that there is a correlation between FMA scores and global efficiency. These findings help us better understand the dynamic brain network of patients with post-stroke when processing MI information. The network properties may provide a reliable biomarker for the objective evaluation of the functional rehabilitation diagnosis of stroke patients.

## Introduction

Stroke, also known as cerebrovascular accident, is a disease of the blood vessels supplying the brain are damaged. It can lead to avascular necrosis or hemorrhage of our brain tissue. Stroke has high morbidity, disability, and mortality rates, 40% of stroke survivors still suffer from various disabilities, and the incidence of stroke increases disproportionately with age. Moreover, aging is a stroke risk factor (Egorova et al., 2019).

Two types of Motor Imagery (MI) can be divided: Kinesthetic Motor Imagery (KMI) and Visual Motor Imagery (VMI). KMI is defined as the thought process of imagining a given movement without any motor output. VMI mainly relies on the visualization of the execution of that movement (Rimbert et al., 2019). MI is regarded as a mental process involving a variety of advanced cognitive functions (Li et al., 2019). The MI-based brain-computer interface (BCI) has been widely used in motor function rehabilitation, motor skill learning, and other fields (Long et al., 2011; Mane et al., 2020; Xu et al., 2021b). Patients with motor cortex damage can get better functional recovery by MI therapy (Xu et al., 2021a). Researchers aim to obtain good performance from MI recognition (Xu et al., 2016, 2021c; Wang et al., 2020). Electroencephalography (EEG), as a method of recording brain activity using electrophysiological indicators, has the characteristics of high time resolution, low cost, and easy operation (Zhang et al., 2015; Xu et al., 2020a). EEG is the most commonly used brain signal for BCI (Xu et al., 2021d). In recent years, EEG-based BCI systems have developed rapidly, and the number of commands that BCI can process has increased from the initial 30 to more than 100 (Xu et al., 2020b). Recently, the measurement precision of BCI first reached the level of the sub-microvolt in amplitude (Xu et al., 2018), which significantly broadened the category of BCI. EEG is also the commonly used brain signal for clinical rehabilitation. Ding et al. (2022) have used transcranial magnetic stimulation and electroencephalography (TMS-EEG) to directly measure cortical responses in aging stroke patients after intermittent theta-burst stimulation (iTBS) and found that iTBS can normalize natural frequency in aging stroke patients, which can be utilized in stroke rehabilitation.

The human brain is a complex network consisting of a large number of interconnected cortical regions. Recently, the brain network method has attracted much attention and has been widely used in decoding related cognitive functions. The main methods of brain networks are effective and functional connectivity. Functional connectivity is an undirected network that represents the coordination mechanism between different neurons (Reid et al., 2019). Effective connectivity is a directed network defined as the direct or indirect influence from one brain function area to another brain function area (Park et al., 2018).

Based on EEG analysis, directed networks have directional information compared to undirected networks. The directed networks can more accurately assess the information flow between brain nodes and better understand the brain's information processing mechanism when performing MI recognition. Directed analysis methods such as granger causality analysis (GCA), partial directed coherence (PDC), and directed transfer function (DTF) have significant advantages in capturing directional coupling between different brain regions (Jastreboff, 1990; Maudoux et al., 2012). Based on the DTF method, Vecchio and Babiloni (2011) have found that the directionality of frontal-parietal EEG synchronization in Alzheimer's Disease (AD) and Amnestic Mild Cognitive Impairment (MCI) is abnormal.

EEG has millisecond-level time resolution, which leads to different network structures corresponding to different stages of the brain processing information. Therefore, the study of time-varying networks helps us to explore the dynamic process of brain information processing in MI recognition and to capture the time-varying connections of cognitive processes. Including time-varying granger causality analysis (tv-GCA), time-varying partial directed coherence (tv-PDC), and adaptive directed transfer function (ADTF) can get different network connection structures in different cognitive procedures (Li et al., 2015; Manomaisaowapak et al., 2015). Li et al. (2016) have used an adaptive directed transfer function to construct a time-varying network of P300 and found that different stages of P300 induce different brain network structures. Based on the ADTF method, Si et al. (2019) have studied the role of the frontal cortex in the decision-making stage and the different network structures in different decision-making stages.

Fugl-Meyer assessment (FMA) is an authoritative method to assess the motor function of stroke patients. It can provide a visual representation of motor function after stroke, and can play an important role in the baseline assessment, as well as monitor and quantify longitudinal changes in motor function (Riahi et al., 2020). FMA is a reliable and effective method for measuring motor dysfunction, a higher score corresponds to better motor function (Saes et al., 2019). All patients have been completed the FMA to ensure the consistency of the FMA scores and the EEG recording. Saes et al. (2021) have used the resting state EEG parameters of stroke patients to predict FMA scores, and they have proved that resting-state EEG parameters can be used as a biomarker for predicting stroke recovery. A challenge associated with this assessment is the availability of trained doctors to conduct the evaluation. The study of biomarkers can estimate that FMA may help to solve the problem.

The network mechanism of stroke patients based on the ADTF method has been studied. The dynamic reorganization and compensation of the brain network have been revealed. The correlation between network properties and FMA scores has been analyzed. Our proposed method provides a new neuroregulatory index for diagnosis and treatment of post-stroke patients.

## Materials and Methods

### Participants

After receiving a detailed explanation of the purpose and potential risks of the experiment, all subjects have provided written informed consent. The study protocols have been approved by the medical ethics committee of Qilu Hospital, Cheeloo College of Medicine, Shandong University. The study is carried out in accordance with relevant guidelines and regulations. Twenty-one right-handed subjects have been recruited in our current study, consisting of seven male patients with left hemiplegic stroke (marked as LS, age 49 ± 12 years); five male patients with right hemiplegic stroke (marked as RS, age 54 ± 8 years); nine male health control (marked as HC, age 45 ± 12 years). All subjects have normal hearing and vision, and no psychiatric drugs are taken for healthy subjects.

### Experimental Procedures

**Figure 1:**
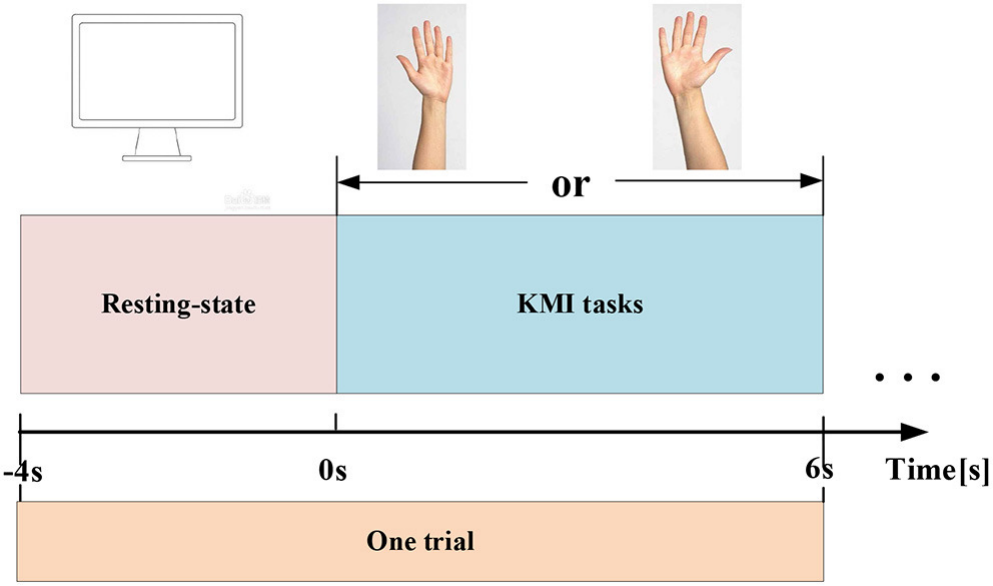
EEG experimental paradigm. One KMI trial includes a 4-s resting state (represented by a blank screen), and a 6-s KMI task (represented by the left or right arrow on the screen).

The experiment is conducted in a separate relatively shielded room. The room is lighted with soft luminance. In addition, during the acquisition of EEG signals, the indoor temperature is maintained at ~21°C by the air conditioner, and the doors and windows are tightly closed to avoid the influence of noise. Throughout the experiment, all subjects are asked to stay relaxed to avoid real hand movements affecting the validity of the data. The experiment adopts the KMI paradigm. Each subject has performed 70 independent experiments, including 30 MIs for each of the left and right hands, 10 actual exercises, and EEG data have been acquired from 64 electrodes. Each KMI trial has a total of 10 s. The first 4 s are resting, and a blank screen appears to remind the subjects to prepare, and the next 6 s are the task state. When the KMI recognition starts, a left or right arrow appears on the screen to remind the subjects to imagine the left-hand or right-hand lifting action. The left-hand or right-hand KMI trials are randomly presented to the subjects. The experimental paradigm is shown in Figure 1.

### Signal Recording

A BrainAmp 67-node amplifier from Brain Products (Australia) has been used to record EEG. All 64 Ag/AgCl electrodes are placed according to the 10–20 international system. The REF electrode between the CZ electrode and the CPZ electrode is used as a reference. In all experiments, the sampling rate is 1000 Hz.

### Data Analysis

**Figure 2:**
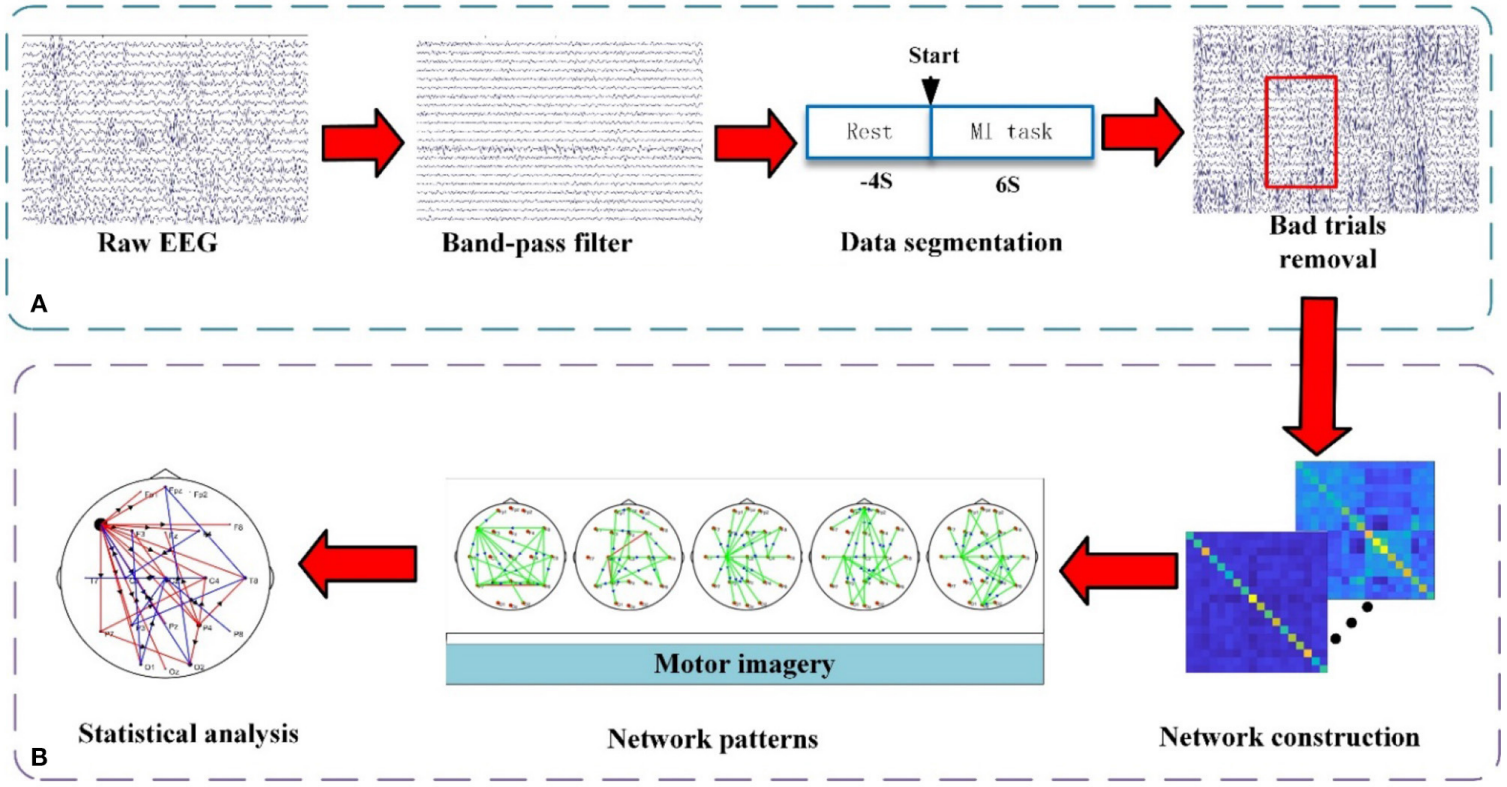
The framework of EEG processing procedure. **(A)** Preprocessing. **(B)** Time-varying network pattern analysis. Between the two electrodes, the connecting edge represents the coupling relationship and the arrow represents the flow direction.

In this study, the preprocessing procedure and analysis procedure are shown in Figure 2, the time-varying network analysis has been performed and the correlation between the global efficiency (GE) and the FMA score has been calculated.

#### Preprocessing

The purpose of preprocessing is to acquire clean EEG data for subsequent analysis. The detailed procedures include 8–30 Hz band-pass filtering, performing reference electrode standardization technique (REST) processing on the filtering data (Yao, 2001; Dong et al., 2017), segmenting data with a time window of [−4 s, 6 s] (0 s corresponds to the stimulus onsets), and removing bad trials [±70 μV as the threshold for ocular artifacts (Li et al., 2019, 2021)]. Then, the data has been down-sampled to 100 Hz. To reduce the influence of the volume conduction between network nodes, 21 electrodes (i.e., Fp1, Fpz, Fp2, F7, F3, Fz, F4, F8, T7, C3, Cz, C4, T8, P7, P3, Pz, P4, P8, O1, O2, and Oz) of the 64 electrodes have been selected to construct the brain functional network.

#### Time-Varying Network Pattern Analysis

For each subject, the preprocessed EEG is used to further construct time-varying KMI networks based on ADTF (Li et al., 2016). Then, the left-hand and right-hand time-varying KMI networks corresponding to each trial are averaged for each subject. Therefore, a time-varying network of two-classes KMI tasks is generated. The detailed description of ADTF in our study is as follows:

##### Time-Varying Multivariable Adaptive Autoregressive Model

For the time series of each subject's trial, the following formula can be used to construct a corresponding time-varying multivariable adaptive autoregressive (tv-MVAAR) model to describe the dataset:

$$\begin{array}{l} X\left(t\right)=\sum_{i=1}^{p}A\left(i,t\right)X\left(t-i\right)+E\left(t\right) \end{array}$$

where *X*(*t*) is the data vector of each trial at time *t*, *A*(*i, t*) denotes the model coefficient matrix estimated by the Kalman filter algorithm (Arnold et al., 1998; Pagnotta and Plomp, 2018), *E*(*t*) denotes the multivariate independent white noise, *p* is the optimal model order automatically determined by the Akaike information criterion (AIC) within the range of 2–20.

$$\begin{array}{l} AIC\left(p\right)=\ln\;\left[\det\left(\varepsilon \right)\right]+2\beta^{2}p/\alpha \end{array}$$

where β is the number of nodes, *p* is the order of the best model of tv-MVAAR, α is the number of sampling points in the time of [−4 s, 6 s] (0 s corresponds to the stimulus onsets), and ε is the corresponding covariance matrix.

##### Adaptive Directed Transfer Function

The time-varying model coefficient matrix *A*(*i, t*) can be transformed in the frequency domain to obtain the transfer matrix *H*(*f, t*) of the time-varying model, which can be further derived *H*_*ij*_(*f, t*) is the directional information flow from the *j*th node to the *i*th node at time *t*. Then, the time-frequency representations of *X*(*t*) and *A*(*i, t*) are described as follows:

$$\begin{array}{r} A\left(f,t\right)X\left(f,t\right)=E\left(f,t\right)\\ X\left(f,t\right)=A^{-1}\left(f,t\right)E\left(f,t\right)=H\left(f,t\right)E\left(f,t\right) \end{array}$$

where $A\left(f,t\right)=\sum_{k=0}^{p}A_{k}\left(t\right)e^{-j2\pi f\Delta tk}$ with the *A*(*t*) denotes the matrix of model coefficients, *X*(*f, t*) and *E*(*f, t*) are the representations of *X*(*t*) and *E*(*t*) in the frequency domain, respectively.

Under the premise of a given frequency *f* and corresponding time point *t*, the ADTF value describes the directional causal interaction from the *j*th node to the *i*th node is normalized and defined as:

$$\begin{array}{l} r_{ij}^{2}\left(f,t\right)=\frac{{\left|H_{ij}\left(f,t\right)\right|}^{2}}{\sum_{m=1}^{n}{\left|H_{im}\left(f,t\right)\right|}^{2}} \end{array}$$

Finally, the ADTF values on the frequency band of interest containing MI-related rhythms at 8–30 Hz are averaged to evaluate the directional information flow of two different nodes (Burianová et al., 2013; Zhang et al., 2018):

$$\begin{array}{l} \Theta_{ij}^{2}\left(t\right)=\frac{\sum_{k=f_{1}}^{f_{2}}r_{ij}^{2}\left(k,t\right)}{f_{2}-f_{1}} \end{array}$$

For each subject, all trials connectivity networks are averaged across all of these artifact-free trials and then induce the final time-varying network model. When exploring the group-wise networks' differences, the time-varying networks of the LS, RS, and HC have been binarily thresholded into the time-varying binary networks with a connectivity cost of 5% to illustrate the time-varying network architectures. The networks have been also statistically compared by using the non-parametric Wilcoxon rank-sum test. Some previous studies have shown that the difference between Θ_*ij*_ and Θ_*ji*_ determines the direction of information flow in time-varying networks (Babiloni et al., 2009; Vecchio and Babiloni, 2011). As ADTF captures the dynamic networks for each time point, and nearby time points have shown highly similar network patterns. In our study, we describe the KMI time-varying networks with a time interval of 1.5 s and reveal the dynamic KMI network mechanism by evaluating the time-varying networks corresponding to different KMI stages.

##### Time-Varying Network Properties

According to the obtained adjacency matrix, Brain Connectivity Toolbox (BCT, http://www.nitrc.org/projects/bct/) has been employed to calculate the GE of all subjects at each time point (Zhang et al., 2020), the time-varying KMI networks are analyzed through graph theory. The GE describes the ability of the brain network to process information. The GE calculation formula is as follows:

$$\begin{array}{l} GE=\frac{1}{n}\sum_{i\in N}\frac{{\sum_{j\in N,j\neq i}\left(d_{\vec{ij}}\right)}^{-1}}{n-1} \end{array}$$

Here, *n* represents the node number, $d_{\vec{ij}}$ represents the shortest characteristic path length, and *N* denotes the set of current network nodes.

#### Correlation Analysis Between Time-Varying Network and FMA

According to the FMA scores, 12 stroke patients have been divided into three classes: severe (FMA: 0–20), moderate (FMA: 20–40), and mild (FMA: 40–60). The 12 patients are ranked from lowest to highest score. The high scores correspond to better motor function, and the low scores correspond to poor motor function. Pearson correlation analysis has been used to explore the potential relationship between each patient's GE and FMA scores to reveal whether the network properties can be used as potential biomarkers to indicate the degree of motor function rehabilitation.

## Results

### Dynamic KMI Network Patterns

**Figure 3:**
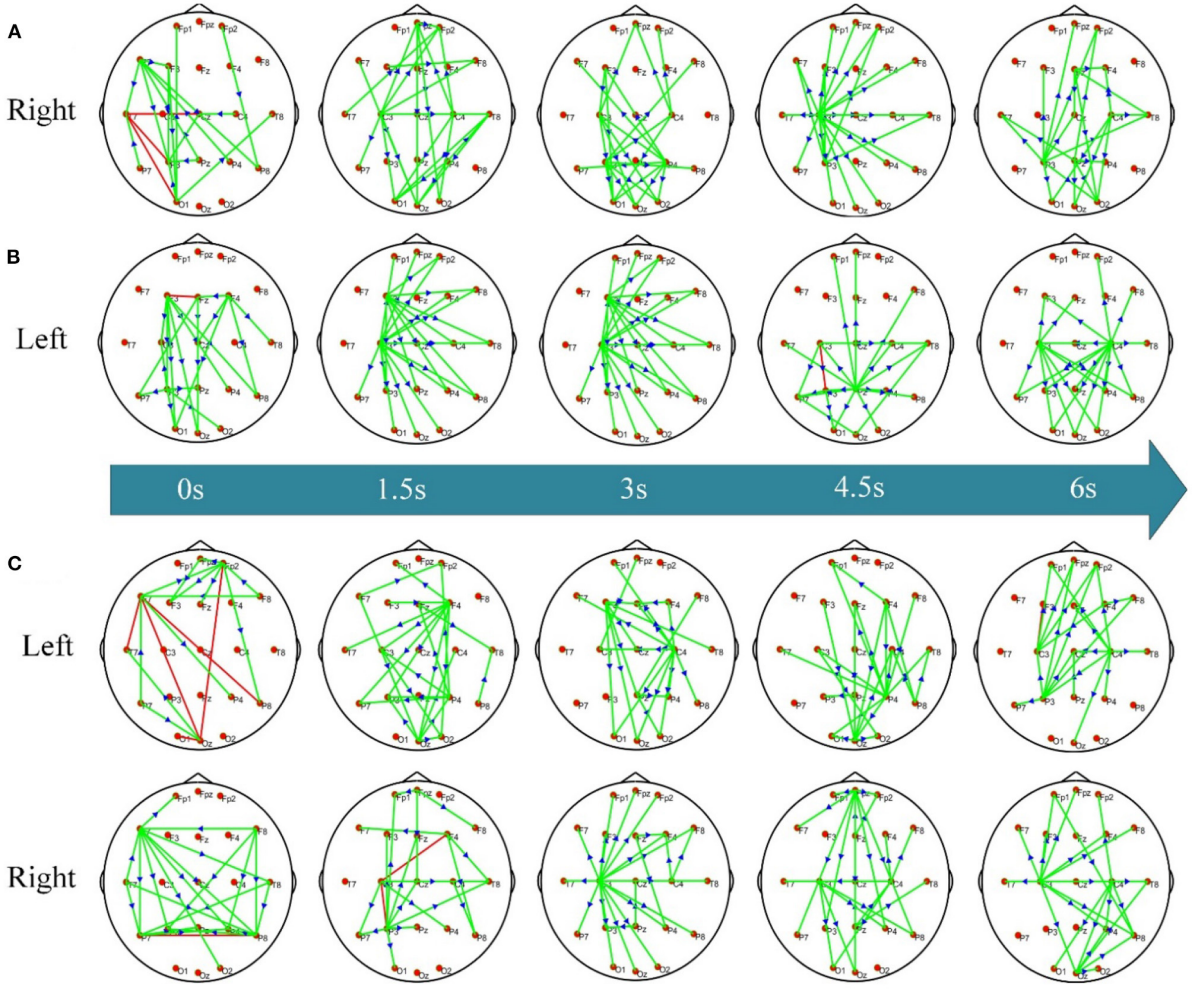
The dynamic KMI network patterns of RS/LS/HC. **(A)** Time-varying network pattern in the right hand of the RS group; **(B)** time-varying network pattern of the left hand in LS group; **(C)** time-varying network pattern of left/right hand in HC group. The connecting edge in the figure represents the coupling relationship between the two electrodes, the red edge represents the two-way connection between the two nodes, the green edge represents the one-way connection between the nodes, and the arrow represents the flow direction between them.

To investigate the dynamic network difference between post-stroke hemiplegic patients and healthy subjects when performing KMI recognition, the ADTF function has been used to calculate the time-varying network matrix of LS, RS, and HC groups in the 8–30 Hz frequency band of interest, and take the sparsity of 5% (i.e., the connection edge with the strongest weight remaining 5%) to display the transient topology. When performing the right-hand KMI tasks, the crucial hubs for the RS subjects (Figure 3A) are located at the contralateral P4 and ipsilateral P3. The motor areas of the stroked hemisphere (i.e., right hemisphere) for the LS subjects (Figure 3B) have been shown the weaker connectivity when executing the left-hand KMI tasks, but the contralateral F3 and C3 electrodes (i.e., at the left hemisphere) extend to the occipital lobe have been shown the stronger connectivity. However, the electrodes C3 or C4 for the HC subjects (Figure 3C) have served as the important hub to control the KMI recognition, and then have transferred to the joint control from bilateral C3 and C4 electrodes.

### Dynamic Network Differences

**Figure 4:**
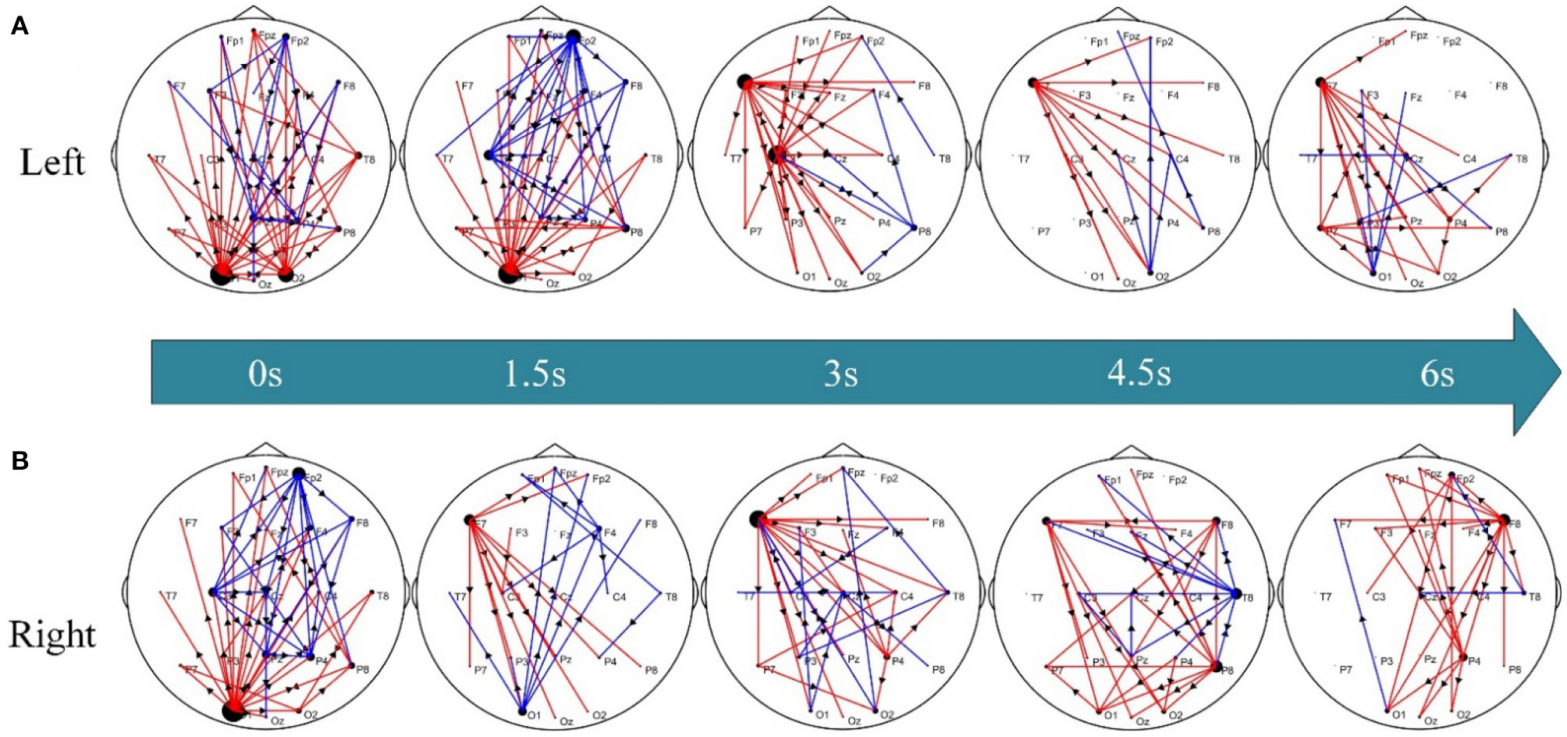
Differential time-varying network topologies between the pairwise groups. **(A)** LS vs. HC groups and **(B)** RS vs. HC groups. Here, the red edge represents the connection edge where LS/RS is stronger than HC, the blue edge denotes the connection edge where HC is stronger than LS/RS, and the arrow indicates the information flow between nodes.

To further explore the differential dynamic network patterns of the time-varying networks between post-stroke hemiplegic patients and healthy subjects, Figure 4 shows the corresponding statistical network topology diagrams at different time points. Compared to HC subjects (Figure 4A), stronger information flow in the LS group has transferred from the occipital lobe (e.g., O1, O2) to the left frontal lobe (e.g., F7); however, these phenomena in the RS group (Figure 4B) have occurred from occipital lobe (e.g., O1) and left frontal lobe (e.g., F7) to the right frontal lobe (e.g., F8).

### Dynamic of the Time-Varying GE

**Figure 5:**
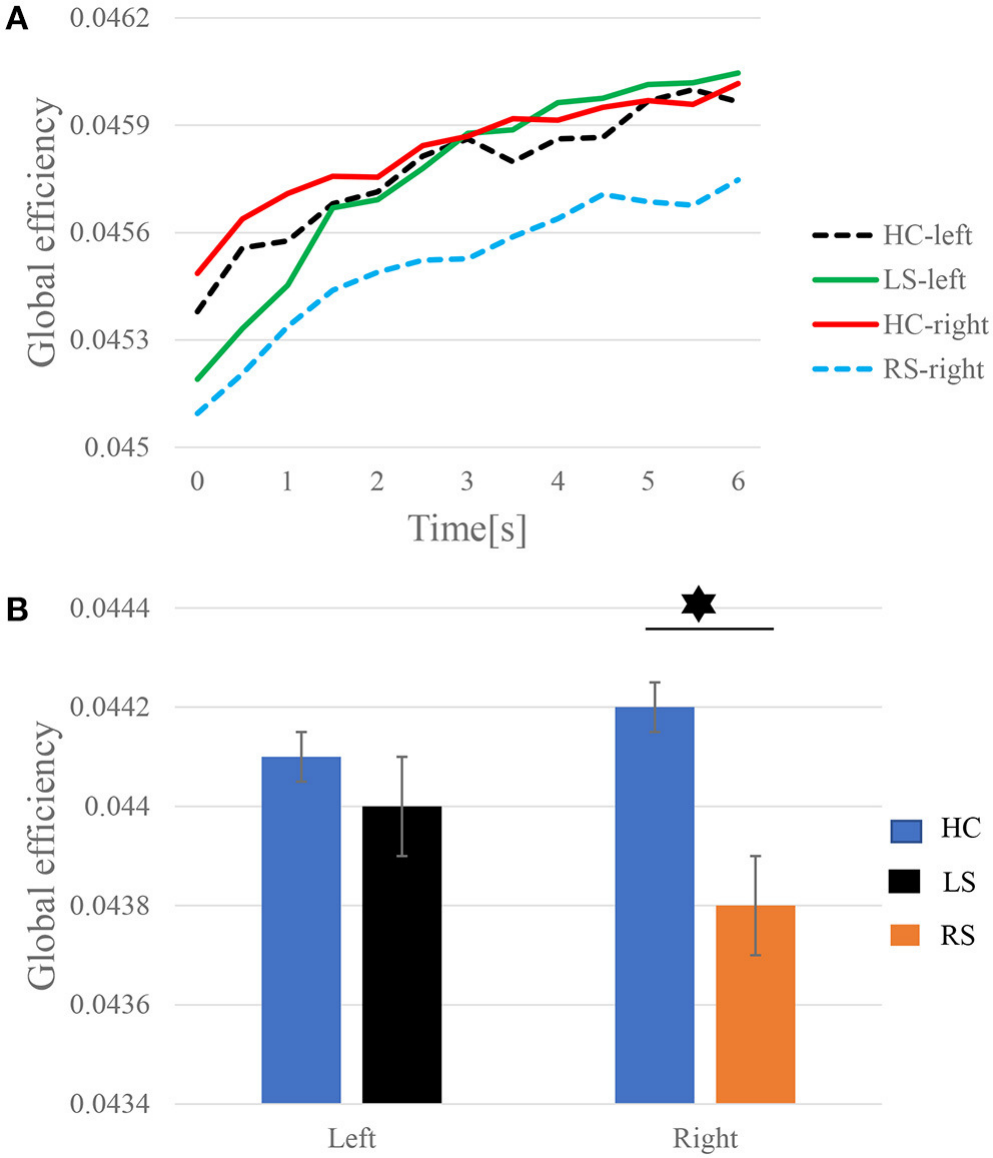
The time-varying GE of left-hand and right-hand KMI recognition. **(A)** Dynamics of the GE. **(B)** Statistics of the average GE. The asterisk represents significant differences in GE between the two groups (*p* < 0.05).

To further explore the connection pattern of the time-varying network, the values of GE at each time point are averaged for the subjects in the three groups of LS, RS, and HC. Figure 5A shows the GE increase along with the progress of the KMI recognition. When performing right-hand KMI tasks, the GE of the HC group is greater than that of the RS group (*p* < 0.05), as shown in Figure 5B.

### Correlation of GE and FMA Scores

**Figure 6:**
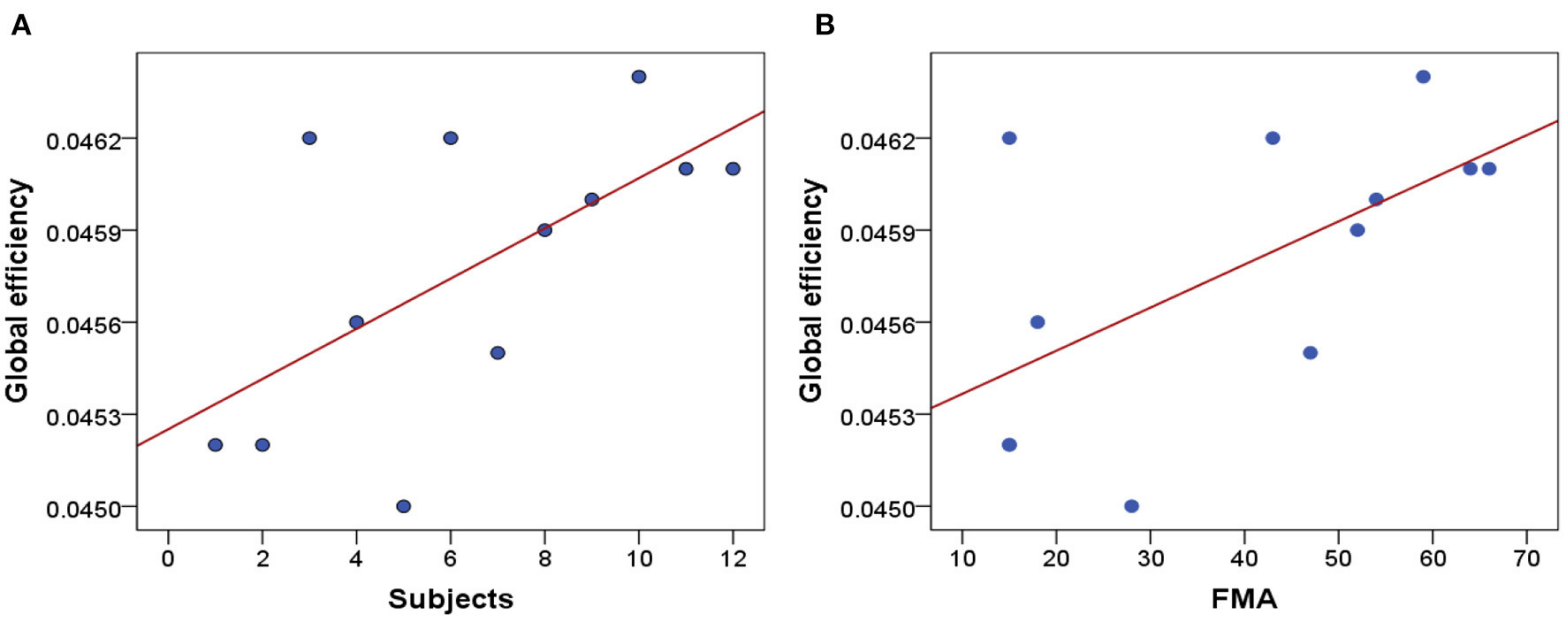
Correlation analysis. **(A)** The average GE of 12 stroke patients. **(B)** Correlation between FMA scores and GE of 12 stroke patients.

Clinically, the higher the FMA scores are responding to the less severe the damage of motor function. Figure 6A shows the average GE of 12 stroke patients, and the x-axis represents the 12 stroke patients who have been ranked in ascending order of FMA scores. The scatter plot of GE and FMA of 12 stroke patients and the positive correlation (*r* = 0.61, *p* = 0.035) are shown in Figure 6B.

## Discussion

Stroke causes damage to the motor functional areas of the brain, which in turn leads to motor dysfunction. Compared with healthy subjects, the functional connections between different brain regions of stroke patients are more complicated in performing KMI. Moreover, the brain processes information very efficiently, which leads to different network structures corresponding to different cognitive stages. To evaluate the network reorganization and compensation of brain function after stroke, the ADTF has been employed to better explore the dynamic network mechanism of post-stroke hemiplegic patients and healthy subjects during the execution of KMI.

Time-varying network topology diagrams under different conditions are calculated to study the interaction patterns between different brain regions of post-stroke hemiplegic patients and healthy subjects. Figure 3 shows the dynamic network patterns of the RS, LS, and HC groups when performing KMI recognition. When the patients with left brain damage perform right-hand KMI tasks, the connection between the motor areas on the stroked left hemisphere and other functional brain areas is enhanced, and the hub node has transferred from node C3 to node C4, as shown in Figure 3A. The enhancement of the bilateral occipital lobe (i.e., P3 and P4) connection is enhanced during the later stage of KMI. These phenomena might further indicate that the contralateral brain areas of the stroked hemisphere have functional compensation, and the ipsilateral non-motor areas that are responsible for the high-level cognition also have functional compensation, such as motor planning and attention (Li et al., 2021). When the patients with right brain damage imagine the left-hand movement, stronger functional connectivity has existed between the frontal and parietal-occipital lobe, while seldom connectivity of the stroked right hemisphere has been observed, as shown in Figure 3B. The phenomena may account for the deficits in performing left-hand KMI tasks and left-hand wrist extension of the LS patients. The frontal and parietal lobe is responsible for the advanced regulation of limb movement. Right brain damage causes human motor dysfunction, the left brain areas increase the response to provide compensation for motor function (Li et al., 2020). Thereafter, the bilateral motor areas C3 and C4 are more connected. Because the brain of stroke patients is damaged, the response pattern and functional connection of the brain are different from healthy subjects. The location and severity of brain damage affect the degree of brain function network remodeling (Arun et al., 2020). For healthy subjects, when performing left-hand and right-hand KMI tasks, the brain function networks appear a network pattern from the opposite side to the bilateral connection, as shown in Figure 3C. When performing the left-hand KMI tasks, the network connection of the right motor areas is enhanced, and then the network connection gradually appears in the bilateral motor areas. During the right-hand KMI procedure, the left motor areas have presented significantly stronger connectivity, which has switched to a bilateral connectivity architecture. During KMI procedure, the brain functional areas involved in healthy subjects include the main motor areas, medial frontal gyrus, parietal lobe, and primary motor cortex (Zhou et al., 2022). The KMI procedure of health subjects mainly responds to the contralateral brain areas (Sharma and Baron, 2013).

The functional compensation and plasticity of the brain after stroke are related to the functional connection difference between stroke and healthy subjects, and are related to the response between different brain regions (Bundy and Nudo, 2019). The study further explores the abnormal networks connection status of stroke patients. Under the premise of the LS group and HC group, the connecting edge of LS is significantly stronger than HC from the occipital lobe to the left frontal lobe, as shown in Figure 4A. At the beginning of the KMI recognition, when the subjects see the prompt instruction, the LS is relative to the HC, the connection of the occipital lobe is stronger at this time. The occipital lobe is the center of the visual cortex (Chu et al., 2021). The damage to the brain motor function areas of stroke patients leads to paying more attention to prompt instructions. Therefore, the patients' attention to action prompt instructions is also a good compensation effect for the motor dysfunction (Rowe et al., 2002). In addition, the stronger connectivity of the occipital lobe plays an important role in improving the performance of SSVEP-based BCI systems (Gao et al., 2018; Sun et al., 2020). During KMI recognition, the functional connections of the LS brain are enhanced from the left frontal lobe (i.e., F7 node) to the bilateral parieto-occipital lobes. The connection of the frontal and parietal brain areas plays an important role in motor planning, decision-making, etc. (Bowling et al., 2020). The frontal lobe is related to the movement of the limbs. Right brain stroked in LS leads to increased connections between the left frontal lobe and other brain regions. The phenomena show that the left brain areas participate in motor planning and regulation as compensation when performing KMI recognition. As shown in Figure 4B, the significantly stronger connection edges of RS have transferred from the occipital lobe and the left frontal lobe to the right frontal lobe compared to HC. Stroke results in the dysfunction of the patients' motor network, more non-injured brain areas and non-motor areas of the damaged brain areas can participate in the completion of KMI recognition (Li et al., 2020). The connection of the brain network of the frontal lobe and the occipital lobe is abnormal, the functional compensation of the brain to the damaged motor network is indicated.

Based on the time-varying networks of the three groups of subjects, the time series of the dynamic GE of the different groups in the KMI stage are shown in Figure 5. The GE is the average efficiency of related brain networks and is usually used to estimate the potential of information transfer among brain regions. As illustrated in Figure 5A, the time-varying network efficiency of the HC, LS, and RS groups has increased along with the execution. When subjects are asked to perform KMI recognition, more advanced cognitive functions in the brain are gradually recruited, so network efficiency gradually increases (Zhang et al., 2016). Throughout the KMI recognition stage, the gradual increase in network efficiency can guarantee the completion of KMI recognition. Sabaté et al. (2004) have found that after left hemisphere stroke, a person's limb movement speed is significantly slowed down, and after right hemisphere stroke, the brain activity during KMI is stronger than that in the left hemisphere stroke. The information transfer rate between brain regions in the RS group is lower than the LS group when performing KMI recognition. LS has stronger brain compensatory and remodeling capabilities. After a stroke, plastic changes occur between different brain areas, the interaction between brain areas is enhanced to compensate for the damaged brain areas. The patients need to activate other brain areas as compensation to complete the KMI recognition. And indeed, when performing the right-hand KMI tasks, the average GE of the HC group is significantly larger than that of the RS group, as shown in Figure 5B.

To further investigate whether the GE is correlated with the FMA scores, we have performed one correlation analysis. As shown in Figure 6B, there is a positive correlation between GE and FMA. The higher the FMA scores, the higher the corresponding global efficiency. It proves that the GE can reflect the severity of clinical motor function damage. We can conclude that GE may be used as a potential biomarker to reflect the severity of motor function damage and objectively evaluate the efficacy of neuromodulation therapy. And it can also be used as a feedback indicator to guide the development of more effective KMI rehabilitation therapies in the future.

Our current study also has some limitations. The number of patients is scarce, and the subjects between males and females are unbalanced. To promote clinical treatment and effective intervention for stroke, more subjects will be recruited, meanwhile, the balanced male and female subjects will be considered for analysis.

## Conclusions

In our study, we have constructed the time-varying KMI networks between post-stroke hemiplegic patients and healthy subjects based on the ADTF method. In post-stroke hemiplegic patients, the connection between the damaged brain areas and other motor areas is weaker when performing KMI recognition. The effective connection between the non-damaged brain areas and other motor areas is stronger. The connection between the frontal-parietal lobe and the occipital lobe is enhanced to provide compensation for motor dysfunction in stroke patients, and FMA scores are closely correlated with GE. These findings allow us to better understand the mechanism of movement disorders in patients with post-stroke hemiplegic. It also shows that the brain network may provide a more reliable quantitative analysis method for the clinical diagnosis and treatment of stroke.

## Data Availability Statement

The raw data supporting the conclusions of this article will be made available by the authors, without undue reservation.

## Ethics Statement

The studies involving human participants were reviewed and approved by the Medical Ethics Committee of Qilu Hospital, Cheeloo College of Medicine, Shandong University. The patients/participants provided their written informed consent to participate in this study.

## Author Contributions

XY, CW, ML, CF, and JLi have designed the experiment. DW and YZ have conducted the experiments. FX, YW, HL, and LJ have conducted a brain function analysis. FX, YW, HL, ZY, and JLe have performed correlation and statistical analysis. ZY, YZ, and JLe have reviewed the manuscript. FX is a major contributor to the writing of the manuscript. All authors contributed to the article and approved the submitted version.

## Funding

The project was supported in part by the Introduce Innovative Teams of 2021 New High School 20 Items Project under Grant No. 2021GXRC071, in part by the Program for Youth Innovative Research Team in the University of Shandong Province in China under Grant No. 2019KJN010, in part by the Natural Science Foundation of China under Grant Nos. 82172535, 61877062, 61977043, 62122059, and 61976152, in part by the Clinical Research Cross-Project of Shandong University under Grant No. 2020SDUCRCB004, in part by the Natural Science Foundation of Shandong Province of China under Grant Nos. ZR2019MA037, ZR2019PF002, and ZR202102200383, in part by the Research Leader Program of Jinan Science and Technology Bureau under Grant No. 2019GXRC061, in part by the Graduate Education and Teaching Reform Research Project of Qilu University of Technology in 2019 under Grant No. YJG19007.

## Conflict of Interest

The authors declare that the research was conducted in the absence of any commercial or financial relationships that could be construed as a potential conflict of interest.

## Publisher's Note

All claims expressed in this article are solely those of the authors and do not necessarily represent those of their affiliated organizations, or those of the publisher, the editors and the reviewers. Any product that may be evaluated in this article, or claim that may be made by its manufacturer, is not guaranteed or endorsed by the publisher.
